# A biocompatible NPK^+Fe+Zn^ slow release fertilizer: synthesis and its evaluation in tomato plant growth improvement

**DOI:** 10.1038/s41598-024-55152-z

**Published:** 2024-02-26

**Authors:** Tahereh Raiesi Ardali, Leila Ma’mani, Mostafa Chorom, Elaheh Motamedi, Mohammad Fathi Gharebaba

**Affiliations:** 1 Department of Nanotechnology, Agricultural Biotechnology Research Institute of Iran (ABRII), Agricultural Research Education and Extension Organization (AREEO), Karaj, Iran; 2 Department of Soil Science, Faculty of Agriculture, Shahid Chamran University of Ahvaz, Ahvaz, Iran; 3 Department of Molecular Physiology, Agricultural Biotechnology Research Institute of Iran, AREEO, Karaj, Iran

**Keywords:** Nanofertilizer, Nanocomposite, Polymer, Slow release, Tomato, Ecology, Plant sciences

## Abstract

Slow-release fertilizers (SRFs) play an essential and necessary role in sustainable agriculture. Using slow-release and environment friendly fertilizers can increase the growth of plants and reduce the loss of nutrients. Considering the deficiency of iron (Fe) and zinc (Zn) in calcareous soils, a slow-release fertilizer was prepared based on the polymeric nanocomposite, which contains NPK, Fe, and Zn. Its potential was evaluated on tomato plant growth by conducting an experiment in a factorial completely randomized design with three replications. Two levels of salinity (2 and 5 ds m^−1^, two types of soil texture) clay loam and sandy loam) and five levels of fertilizers were examined in the experiment. To this, the graphene oxide-chitosan coated-humic acid@Fe_3_O_4_ nanoparticles (Fe_3_O_4_@HA@GO-Cs), and the graphene oxide-chitosan coated-ammonium zinc phosphate (AZP@GO-Cs) were used as Fe and Zn sources, respectively. Then, the optimal Fe and Zn fertilizers in the presence of urea, phosphorus, and potassium slow- release fertilizers (SRF) were investigated under greenhouse conditions. The results indicated that the best improvement in growth and nutrient uptake in plants was achieved by using the SRF. Notably, in the shoots of tomato plants, the nitrogen, phosphorus, potassium, Fe, and Zn concentration increased by 44, 66, 46, 75, and 74% compared to the control. The use of nanofertilizer can be an effective, biocompatible, and economical option to provide Fe and Zn demand in plants.

## Introduction

Micronutrients are essential components of enzymes and proteins and are vital for increasing the yield of agricultural products and improving the nutritional quality of food^1^. Iron (Fe) is one of the essential elements for plant growth, vital for photosynthesis reactions. This element also plays a fundamental role in the activation of several enzymes that are involved in the reduction and fixation of nitrogen, energy transfer, participation in the synthesis of ribonucleic acid (RNA), and lignin formation^2^. In the second place, Zn is the second most abundant transition metal in organisms. Zinc is present in the structure of transferases, oxidoreductases, lyases, hydrolases, ligases, and isomerases. Also, several reactions are catalyzed by Fe and Zn elements in plants^2^. Deficiency of low-use elements in calcareous soils of densely populated areas of the world with high pH and high bicarbonate content, low organic matter, drought, and salinity stress, are widespread^3^.

Nanotechnology is a suitable approach to improve the nutritional efficacy of agricultural fertilizers, besides their reduced consumption and environmental pollution, than agricultural fertilizers^4^. Many reports confirm the positive effects of Fe NPs on the growth parameters in several plants such as tomato^5^, wheat^6^, soybean, alfalfa^7^, and soybean^8^. These reports showed that Fe NPs could improve the morphological, physiological, biochemical, and plant performance characteristics. Graphene oxide-based materials can provide a very suitable surface for loading and transporting micronutrients due to their high specific surface area and physicochemical properties. The efficient electrostatic interactions of these nanomaterials in the structure of SRF may provide appropriate conditions for binding and loading various cargoes such as micronutrients. Kabiri et al.^9^ used graphene oxide as a carrier in the SRF structure for the delivery of Znand copper nutrients and stated that this fertilizer has satisfactory results compared to the commercial zinc sulfate and copper sulfate granules.

Considering the urgent need for food security and sustainable development, the importance of introducing effectively formulated fertilizers is nowadays very noticeable and severe. Therefore, many efforts have been focused on offering environmentally benign coatings to produce the SRFs^10,11^. Kumar et al.^12^ investigated the combination of PVAs starch-based film polymer with carbon nanofibers to release the Cu–Zn NPs .They stated that the use of these NPs increased the germination of chickpea seeds by 96%. Also, these NPs increased the amount of fresh biomass, water percentage, chlorophyll, and plant protein.

Many of these coating formulations derived from natural materials have significant advantages, including easy access, cost-effective production, and proper biodegradability compared to synthetic materials^13^. Several natural polymers including starch^14^ , cellulose^15^, and chitosan^16,17^ have been investigated. For example, chitosan (CS) is a linear polysaccharide and a derivative of glucan, which is low-cost, biodegradable, non-toxic, and suitable for producing controlled-release fertilizers^18^. Hydrogels prepared with chitosan nanoparticles (CS NPs) through the polymerization of methacrylates can produce coated NPK fertilizers^16^. Abdel-Aziz et al.^17^ showed that the application of CS NPs increased the absorption of NPK in the rice plant. It also increased the grain yield of crops by 49% compared to the conventional NPK fertilizer and reduced the growth cycle of rice. Therefore, using chitosan NPs caused a slower release of elements from the fertilizer.

In the findings of recent studies of water polymer latexes containing starch-g-poly reinforced with NCNP and superabsorbent composites for the synthesis of slow-release fertilizers^19^. The efficiency of synthesized SRF samples in greenhouse experiments on tomato plant production was confirmed. Therefore, based on the mentioned and our previous experience in plant nutrients and the application of nanoparticles in plant growth^20,21^, herein, the goal was the synthesis of NPK^+Fe+Zn^ slow-release fertilizer using of the polymeric nanocomposite coating as a carrier for Fe and Zn nutrients required for plant. The efficiency of the Fe and Zn slow-release fertilizers was investigated in the growth of tomato plants in greenhouse conditions and compared with traditional fertilizers.

## Materials and methods

Zinc nitrate (Zn(NO_3_)_2_), sodium nitrate, chitosan (Cs), acetic acid (AA),and diethylenetriaminepentaacetic acid (DTPA), were also purchased from Sigma-Aldrich (USA). Sulphuric acid (H_2_SO_4_ (98%), Hydrogen peroxide H_2_O_2_ (30%), hydrochloric acid (HCl, 37%), butyl acrylate (BA), corn starch, ammonium persulfate (APS, 98%), styrene, and tween 80 were purchased from Sigma-Aldrich (USA)*.* Diammonium phosphate ((NH_4_)_2_HPO_4_), and potassium permanganate were purchased from Merck. Raw natural char was obtained from Kohbanan region of Kerman, Iran.

### Synthesis of graphene oxide-based iron and zinc nanoparticles

#### Synthesis of graphene oxide (GO)

Graphene oxide (GO) was synthesized using the modified Hummer’s method^22^. For this purpose, 5 g of graphite and 100 mL of H_2_SO_4_ were mixed in a beaker. Then 5 g of sodium nitrate was added to it, and its temperature was brought to 0 °C in an ice bath. After that, 20 g of KMnO_4_ was gradually added to it at a time interval of 2 h until the solution turned green. After that, the temperature of the reaction mixture was increased to 35 °C and then stirred at the same temperature for two hrs. Next, 115 ml of deionized water (DW) was added to the reaction vessel, and heated at 90 °C for 30 min. The reaction was stopped by adding 350 ml of DW and 40 ml of H_2_O_2_ (30%) and placed in an ultrasonic bath for one hrs. The solid residual was centrifuged and thoroughly washed with DW. Finally, the resulting brown solid material was dried in a vacuum oven at 70 °C for 24 h.

#### Graphene oxide-chitosan coated ammonium zinc phosphate (AZP@GO-Cs)

To prepare AZP (zinc ammonium phosphate), a 0.3 M solution of Zn(NO_3_)_2_ (zinc nitrate) was added to a 0.3 M solution of (NH_4_)_2_HPO_4_ (diammonium phosphate) at room temperature (RT). The pH of the solution was adjusted to about nine by adding NH_4_OH 28%, and the reaction was allowed to proceed for 12 h at RT under vigorous stirring. Then, it was left for another 24 h, then the illiquid material was dried in a vacuum oven^23^. After that, 0.50 g of GO was dispersed in DW using an ultrasonic bath for 20 min and added dropwise to a clear solution containing 5 g of Cs in 50 ml of 10% AA, and mixed slowly for 12 h to obtain the GO-Cs coating material. In the end, 5 g of AZP was mixed with 500 mg of GO-Cs, and after that, the AZP@GO-Cs were dried at RT.

#### Graphene oxide-chitosan coated Fe_3_O_4_@HA NPs (Fe_3_O_4_@HA@GO-Cs)

Humic acid (HA, 3 g) was dissolved in 50 ml of water, and pH adjustment to 3 was done using 37% HCl*.* Then, Fe_3_O_4_ NPs (2 g) dispersed in DW (50 ml) with sonication, and Fe_3_O_4_ NPs were added to the humic acid solution. The solid residue was washed several times with distilled water, centrifuged, and dried under vacuum at 30 °C. In the next step, 0.50 g of GO in DW was sonicated for 20 min, and 5 g of Cs in 50 ml of 10% AA was added to it, and allowed to stir for 12 h. Then 5 g of Fe_3_O_4_@HA NPs were mixed with GO-Cs, and the Fe_3_O_4_@HA@GO-Cs were cast on glass plates and air-dried overnight.

### Preparation of the SRF by the rotary drum coating method

#### Preparation of latex coating material

To prepare the latex formulation, the NCNPs were synthesized^24^. For this purpose, chemical oxidation of NC powder was performed. Briefly, this powder (0.5 gr) was added to H_2_SO_4_ (50 ml), followed by KMnO_4_ (2 g) and mixture, stirred in an ice bath (2 h). Then the mixture was stirred for another 1 h at 35 °C. After that, 150 ml of DW was added to the reaction vessel, and placed in an ice bath. After that H_2_O_2_ solution (10 ml) was poured into the mixture, and the sediments were centrifuged, washed with DW, and dried at 70 °C. The water-based polymer latex coating precursor was prepared using an emulsion polymerization reaction, and in the synthesis of this fertilizer, starch, styrene monomers, and butyl acrylate was used. Briefly, 5 g of starch was mixed with 60 ml of distilled water with a mechanical stirrer for 30 min. Afterward, natural coal nanoparticles (NCNPs) were dissolved in 10 ml of deionized water and dispersed for 30 min with an ultrasonic device and added to the reaction material. The reaction was stirred for 30 min. A non-ionic emulsifier containing 0.4 g of tween 80 or 1 ml of nonylphenol ethoxylate (KENON 6) and deionized water (20 ml) was added to the reaction and stirred for 10 min. Next, styrene and butyl acrylate were added to the container. Then ammonium persulfate (APS, 0.2 g) in DW (20 ml) was added to a solution contain styrene and butyl acrylate. Next, the reaction temperature was raised to 85 °C and stirred for 3 h to complete the polymerization reaction^19^.

#### Preparation of Biocompatible N, P, K SRFs

Urea, phosphorus, and potassium granules (1 kg) were poured separately into a rotary drum. Next, the previously prepared latex material was sprayed onto the surface of fertilizers that was rotating at 60 rpm speed, and heated at 85 °C for 20 min until providing the granules. The fertilizer is evenly covered, and the water is heated to evaporate completely. In all samples, a coating formula (500 ml) was added to fertilizer granules^19^.

### Measurement of soil characteristics and application treatments

#### Soil sampling and determination

To carry out this research, surface soil samples (depth 0 ˗ 30 cm) were collected from the fields of Alborz province located in Karaj, and after air-drying, the soil was passed through a 2 mm sieve and some of their characteristics were determined (Table 1). The soil texture by hydrometer method^25^; soil reaction (pH) in the saturated extract using a glass electrode^26^; the electrical conductivity of the saturated extract (EC) by a conductivity meter^27^; the concentration of extractable Zn (using DTPA as an extracting agent) by atomic absorption^28^; total N content using Kjeldahl digestion^29^; soil P content by Olsen method^30^; and absorbable K by ammonium acetate method^31^ had been measured.

**Table 1:** The characteristics of the studied soil sample.

Sample	Clay loam	Sandy loam
pH	7.3	7.6
EC (dS m^−1)^	1.73	2.07
Available iron (mg kg^−1^)	3.9	1.6
Available zinc (mg kg^−1^)	1.56	0.56
Available potassium (mg kg^−1^)	145	125
Available phosphorus (mg kg^−1^)	4.8	3.5
Nitrogen (%)	0.174	0.054
Organic matter (%)	1.69	0.7
Soil texture	Clay loam	Sandy loam
Clay (%)	36.4	12
Silt (%)	29.5	14
Sand (%)	34.1	74

#### Cultivation of tomato in greenhouse

Before the cultivation of tomato in the greenhouse, fertilizers were mixed with 2 kg of soil (clay/loam, sandy/loam). Vigorous tomato seedlings were used for cultivating the pots of Cherry tomatoes (Solanum lycopersicum var. cerasiforme). The relative humidity in a greenhouse tomato production was 40%. The temperature of the greenhouse for the day–night cycles was maintained between 24 and 18 °C, and the light–dark cycles was 12—12. Tomato plants were irrigated to 80% of field capacity. By adding NaCl and CaCl_2_ to the irrigation water, salinity was maintained up to the salinity level of 5 (dS/m).

### Fertilizing material and fertilization treatments

To investigate the effects of application of the synthesized fertilizers on the tomato plant growth, an experiment including five different fertilizers and two levels of salinity (2 and 5 ds m^−1^ (and two different textures) clay/loam and sandy/loam(, was carried out in research greenhouse with three replications. Five fertilizer treatments include: (1) Commercial fertilizer: CF, equal to the conventional used dosage, (2) half of the amount of CF used: CF/2, (3) Slow-release fertilizer: SRF, equal to the conventional used dosage, (4) half of the amount of SRF used: SRF/2, (5) no fertilization: Control.

The fertilizer samples were prepared according to the tomato fertilizer recommendation. For CF the amounts of urea, DAP, and potassium sulfate fertilizers were 350 kg ha^−1^ and 160 kg ha^−1^, and 250 kg ha^−1^, respectively. Also, the SRF samples were prepared in such a way that the amount of N, P, and K in them was equivalent to the related CF samples. For Zn fertilizers, zinc sulfate (ZnSO_4_.7 H_2_O) and 50 kg ha^−1^ of AZP@GO-Cs were used, as commercial and slow-released Zn. For Fe fertilizer, 341 kg ha^−1^ of iron sulfate (FeSO_4_.H_2_O) and 286 kg ha^−1^ of Fe_3_O_4_HA@GO-Cs, as commercial and slow-released Fe. All tests were done in three replications. In this study, different treatments of the SRFs of N, P, K, Fe, and Zn were provided using different portions of the biocompatible NPK^+Fe+Zn^ SRFs, before planting.

### Statistical analysis

Statistical analysis of the variance for factorial design was performed with the SPSS version 16.0 statistical software. All experiments were tested three times. Differences among treatments were evaluated using Duncan’s multiple range tests at a significance level of *p* < 0.05.

### Ethics approval

It is declared that the present study comply with relevant institutional, national, and international guidelines and legislation. Herein, tomato cultivation was done and our national research institute (ABRII) where this study was done in, follows the mentioned national and international legislation.

## Results and discussion

### Characteristics of the nanocomposites

**Figure 1:**
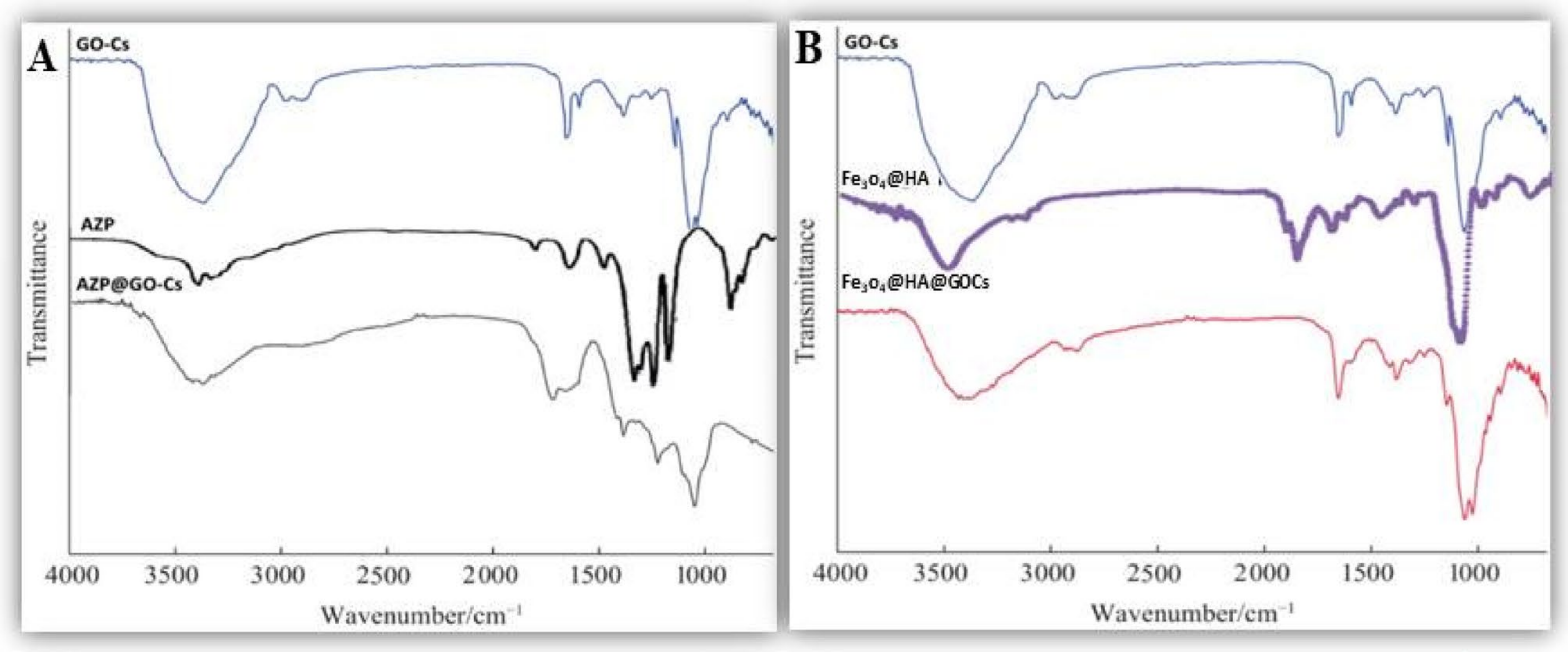
(**A**) FTIR spectra of GO-Cs, AZP, and AZP@GO-Cs; and (**B**) FTIR spectra GO-Cs, Fe_3_O_4_@HA, and Fe_3_O_4_@HA@GO-Cs.

The structures of GO-Cs, AZP, AZP@GO-Cs, Fe_3_O_4_@HA@GO-Cs, and NPK@polymer nanocomposite were confirmed by FTIR, and the results are presented in Fig. 1A. In the spectrum of GO-Cs and AZP (Fig. 1A), the absorption peaks at 3200–3400 cm^−1^ peaks related to hydrogen bonds between free hydroxyl groups of GO-Cs surface and AZP occur. This type is made from the OH functional groups of alcohols, and phenols with the hydrogen group present in amines or alcohols. In the case of AZP@GO-Cs, the peaks at 1000–1300 cm^−1^ can be related to C-O functional groups. In addition, absorption peaks at 1025 and 635 cm^−1^ are observed, attributed to the asymmetric stretching of the P-O bond and the vibration of the Zn–O bond, respectively (Fig. 1A).

**Figure 2:**
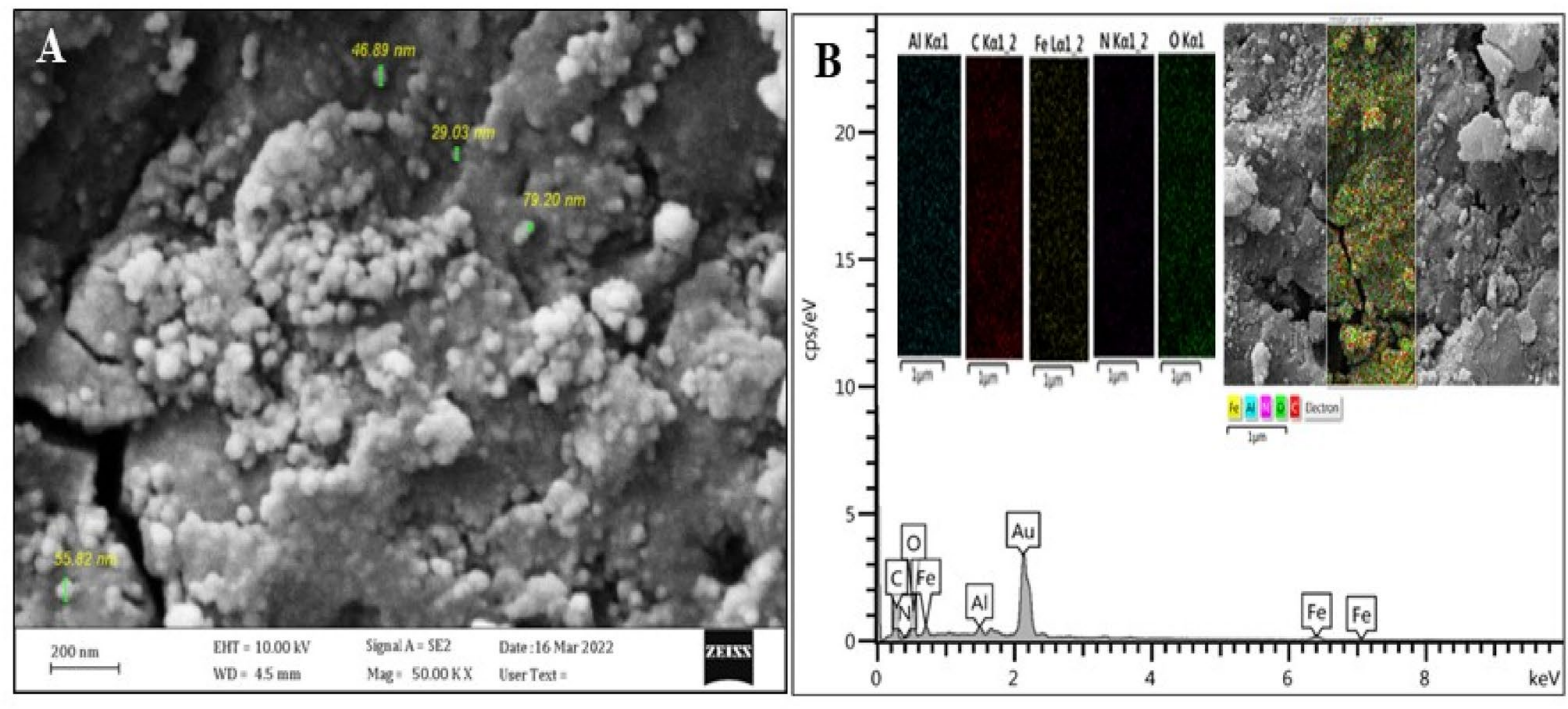
(**A**) FESEM image; and (**B**) EDS spectra and mapping of the elements for Fe_3_O_4_@HA@GO-Cs insert in B.

The structures of GO-Cs, Fe_3_O_4_@HA,and Fe_3_O_4_@HA@CS-GO were confirmed by FTIR, and the results are presented in Fig. 1B. The spectra related to GO-Cs have specific peaks of (-CH) groups that show vibrations of 2950–3000 cm^−1^. Also, C–H=C groups can be seen at the wavelength of 2700–2800 cm^−1^ and C=C vibration at 1400–1600 cm^−1^. The spectra of humic acid-iron coated with chitosan-nanographene oxide GO-Cs have specific peaks of carboxylic acid groups (C=O. in the spectrum of 1000–1300 cm^−1^, they show the peaks of Fe–O and C–O groups (Fig. 1B). Scanning electron microscopy was used to determine the morphologies of the coated NPK fertilizers with nanocomposite latex,^19^ and AZP@GO-Cs. Also, the surface morphology and particle size of the Fe_3_O_4_@HA@GO-Cs were observed by SEM (Fig. 2). The average particle size of Fe_3_O_4_@HA@GO-Cs nanoparticles is about 43.53 nm.

Adding chitosan-nanographene oxide to Fe_3_O_4_-HA creates crosslinks of amino groups in chitosan with Fe_3_O_4_. Co-deposition of GO with chitosan forms a layer of transparent coating on Fe_3_O_4_-HA. Based on this, it can be said that the presence of chitosan and nanographene oxide in the fertilizer structure delays the release of iron. Due to their chemical structures, graphene oxide (GO) and chitosan (CS) can interact through epoxy and amine groups, respectively.

The use of graphene oxide in nanocomposites blocks some interconnected pores of CS and also reduces chemical diffusion and increases the mechanical strength of the film^32^.

### Evaluation of NPK^+Fe+Zn^ slow release fertilizer (NPK^+Fe+Zn^ SRF)

#### Vegetative properties of tomato affected by NPK^+Fe+Zn^ SRF

Analysis of variance (Table 2) showed that salinity, soil texture, and fertilizer significantly affected shoot length, dry weights, and shoot fresh, greenness, nitrogen, potassium, phosphorus, iron, and zinc concentration in shoots tomato plants. The interaction effect of salinity × texture (S × T) was significant for greenness and potassium concentration (Table 2). The interaction effect of salinity × fertilizer was significant only for potassium concentration. The interaction effect of S × T × fertilizer was significant for plant height, shoot fresh weight, shoot dry weight, greenness, N, Fe, and Zn concentration in shoots tomato plants (Table 2).

**Table 2:** Analysis of variance of plant height, shoot fresh weight, shoot dry weight, SPAD reading, N, P, K, Fe, and Zn in the aerial part of tomato plants.

Source of variation	df	Plant height (cm)	Shoot fresh weight (g plant ^−1^)	Shoot dry weight (g plant ^−1^)	SPAD Reading	N (%)	P (%)	K (%)	Fe )mg kg^−1^)	Zn (mg kg^−1^)
S	1	199.546**	282.057**	3.225**	277.264**	0.876**	0.009**	1.365**	9990.277**	1309.001**
T	1	187.267**	106.267**	1.383**	205.646**	0.602**	0.003*	0.782**	7306.274**	836.491**
Fertilizer	4	198.807**	723.956**	7.711**	203.215**	1.096**	0.008**	0.883**	15,141.616**	2286.382**
S × T	1	13.348^ ns^	3.422 ns	0.035 ns	25.168*	0.060 ns	0.001 ns	0.089*	42.943 ns	7.245 ns
S × fertilizer	4	6.665 ns	1.217 ns	0.016 ns	7.961 ns	0.015 ns	0.000 ns	0.036*	115.672 ns	12.515 ns
T × fertilizer	4	1.583 ns	1.188 ns	0.011 ns	1.361 ns	0.004 ns	0.001 ns	0.004 ns	171.037 ns	14.661 ns
S × T × fertilizer	4	13.514 **	23.957*	0.236*	18.960*	0.047*	0.001 ns	0.004 ns	343.868*	56.371*
Error	40	3.557	8.903	0.082	5.590	0.017	0.001	0.014	130.728	17.897
CV (%)		4.83	11	8.86	6.18	6.03	19.2	5.7	8.57	7.23

^a^Salinity: s and Soil Texture t.

*, **: Significant at the 5% and 1% levels of probability, respectively, and ns: Not Significant.

The mean comparison of interaction effect (Table 3) showed that SRF in non-saline soil among fertilizers had the highest amounts for every investigated trait. Mean comparison for S × T × fertilizer interaction (Table 3) indicated that the highest plant height (47 cm) was obtained with SRF application in non-saline soil conditions. The results showed that salinity significantly reduced the plant height of tomatoes. Mean comparison for S × T × fertilizer interaction (Table 3) showed the highest shoot fresh weight and shoot dry weight (44.31 g, 4.94 g) in slow-release fertilizer.

**Table 3:** Mean comparison of interactive effect of fertilizer, salinity, and soil texture on growth traits of tomato.

Treatments	Factors			Means			
	Texture	Salinity	Fertilizer	Plant height (cm)	Shoot fresh weight (g plant^−1^)	Shoot dry weight (g plant^−1^)	SPAD reading
1	T1	S0	Control	36.15^ef^	20.30^gh^	2.53^gh^	37.95^dg^
2			CF/2	37.69^cf^	24.38^fg^	2.95^fg^	39.20^cg^
3			CF	43.47^b^	32.37^b-d^	3.79^b-d^	45.47^b^
4			SRF/2	40.44^b-d^	30.72^ce^	3.60^ce^	42.04^b-d^
5			SRF	47.05^a^	44.31^a^	4.94^a^	49.40^a^
6	T1	S1	Control	29.59^gh^	18.36^hi^	2.25^hi^	31.23^hj^
7			CF/2	35.84^fg^	24.02^eh^	2.88^fh^	37.20^gi^
8			CF	38.43^ce^	26.76^eg^	3.20^ef^	39.60^cg^
9			SRF/2	38.28^ef^	30.47^df^	3.53^ef^	40.09^fh^
10			SRF	40.26^bc^	36.78^bc^	4.20^bc^	40.95^b-d^
11	T2	S0	Control	32.30^h^	17.92^hi^	2.26^hi^	33.15^ig^
12			CF/2	34.44^ef^	21.93^fg^	2.67^fg^	35.17^eh^
13			CF	38.52^ce^	25.53^eg^	3.08^ef^	39.42^cg^
14			SRF/2	35.91^ce^	27.21^ce^	3.20^de^	36.67^cf^
15			SRF	40.67^b-d^	35.42^b^	4.06^b^	41.69^cf^
16	T2	S1	Control	29.28^h^	13.52^i^	1.7^6i^	30.31^j^
17			CF/2	30.36^h^	18.50^hi^	2.28^h^	31.23^ij^
18			CF	36.98^df^	24.99^fg^	3.00^fh^	37.98^dg^
19			SRF/2	31.10^h^	23.35^fh^	2.75^fh^	31.90^ij^
20			SRF	41.16^bc^	36.74^b^	4.19^b^	42.64^bc^

Commercial fertilizer: (CF), half the amount of commercial fertilizer used: (CF/2), slow-release fertilizer: (SRF), half the amount of slow release fertilizer used: (SRF/2), non-saline soil: (S0), saline soil: (S1), clay loam: (T1), and sandy loam: (T2).

The shoot fresh weight and shoot dry weight increased (1.18 times and 95%) compared to the control. Application of slow release fertilizer increases plant growth. The increase in tomato plant growth can be attributed to more efficient uptake, mobility, and iron release from nanofertilizers due to their small size, large surface area, and bioavailability^33^. Khalid et al.^34^ stated that the application of iron and zinc nanofertilizers increased the dry biomass of plants compared to control plants. For Fe_3_O_4_ NPs-based fertilizers, the dry weight of plants increased by 52, 64, 70 and 72% compared to the control at concentrations of 10, 20, 30, and 40 ppm, respectively. Using these nanoparticles increased the fresh biomass, which also increased the dry biomass. Also, the slow- release of zinc in the soil increases the absorption of zinc by the plant.

Our results showed that salinity decreased the tomato plant growth. The reduction of plant growth in saline soils depends on various factors. Saline soils have many adverse effects on plant growth. Among these factors, we can mention the nutritional imbalance, specific ionic effects (salt stress), low osmotic potential of the soil solution (osmotic stress), or a combination of these factors^35^.

Many studies have been conducted on the effect of salinity on plant growth, including the study conducted by Tzortzakis et al.^36^ on tomato (Solanum lycopersicum Mill) who reported that vegetative growth and yield were reduced under salinity treatment at high concentrations. Stamatakis et al.^37^ and Psarras et al.^38^ also stated that increase in salinity decreased the height of the plants.

**Figure 3:**
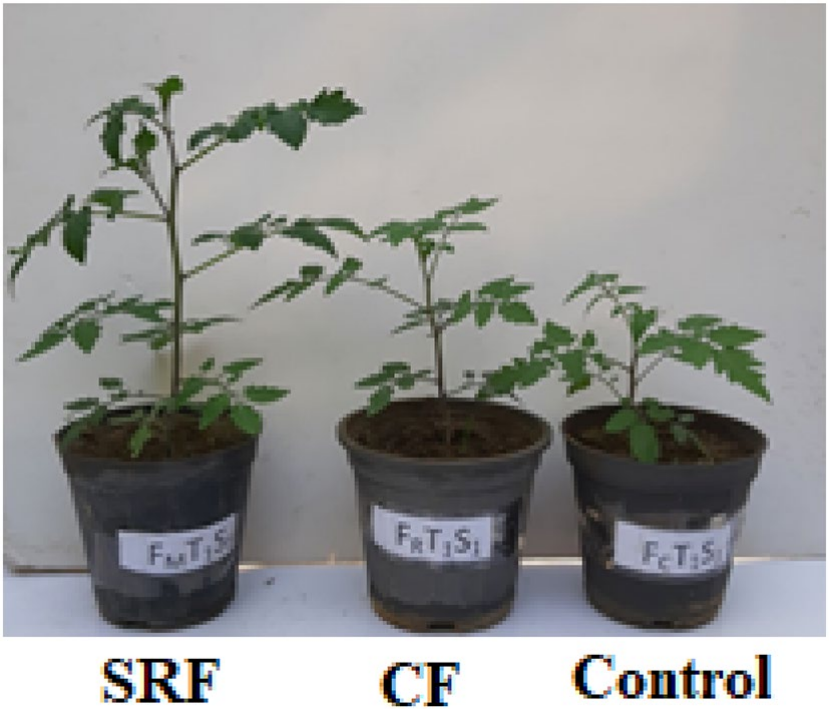
An image of the application of slow-release fertilizers in the soil in the greenhouse.

Nanofertilizers increased the content of chlorophyll. Nitrogen increases the concentration of stromal and thylakoid proteins in the leaf and activates the formation of photo-synthetically active pigments by increasing the chloroplast during leaf growth^39^. In addition, phosphorus facilitates the biosynthetic and biochemical characteristics of pigment molecules^40^. Figure 3 shows that the use of slow-release fertilizers increased the growth of plants compared to the control samples.

Salinity stress reduced photosynthetic pigments. Mean comparison for S × T × fertilizer interaction (Table 3) indicated that the highest amounts of chlorophyll (49.4) was recorded under slow- release fertilizer application in non-saline soil conditions. The reduction in chlorophyll synthesis in saline conditions is due to an increased ethylene production^41^. Also, the reduction in chlorophyll is due to the formation of enzymes such as chlorophyllase. Salinity affects N assimilation and reduces the content of chlorophyll^42,43^.

### The effect of the application of treatments on the total nitrogen of aerial organs

**Figure 4:**
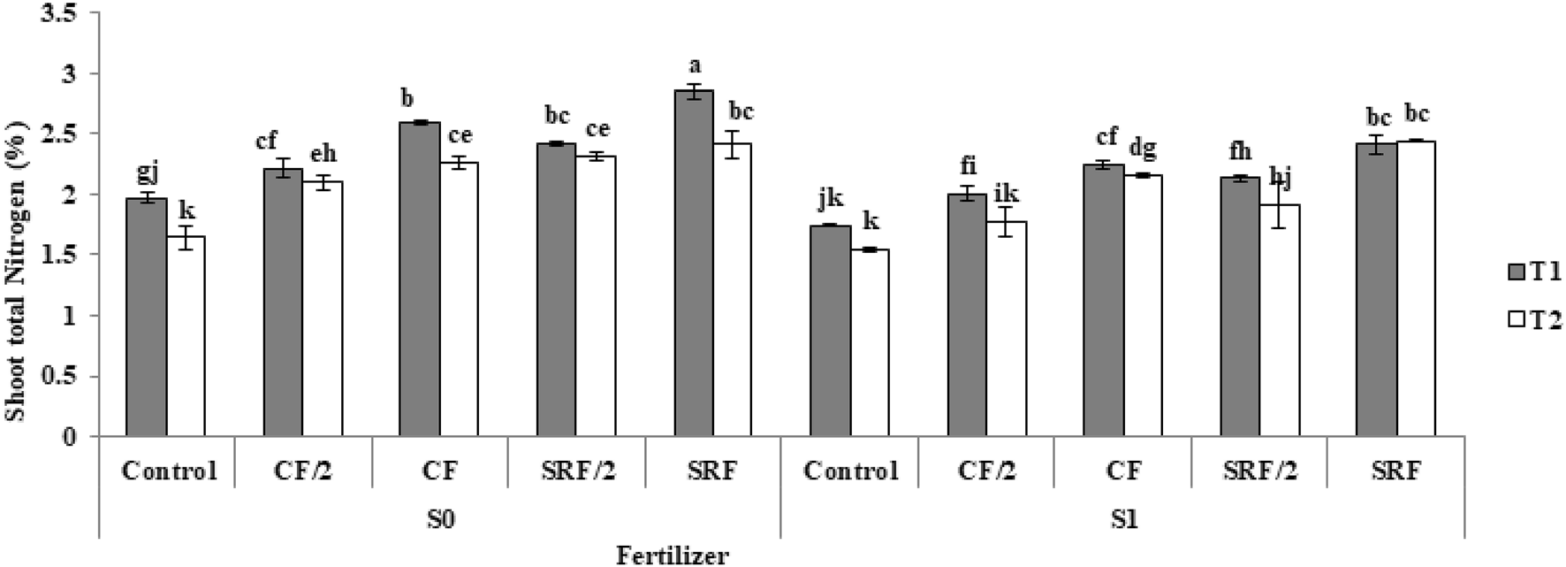
The effect of treatments on total N. The commercial fertilizer: (CF), half the amount of commercial fertilizer used: (CF/2), slow- release fertilizer: (SRF), half the amount of slow release fertilizer used: (SRF/2), non-saline soil: (S0), saline soil: (S1), Clay loam: (T1), Sandy loam: (T2).

The effect of nanofertilizers on N concentration in the aerial part of tomato plants was significant (*p* < 0.05). A similar trend of increasing the percentage and amount of N in the aerial part of the plant can be seen with the use of slow-release fertilizers and commercial fertilizers in comparison with the control, but this increase is more in slow-release fertilizers. The highest amount of total N was observed in the slow-release fertilizer treatments. The highest N concentration in the aerial organs was 2.85%, which was 44% higher than the control (Fig. 4).

Plant nitrogen uptake is the basis of plant photosynthetic activity and, therefore, is closely related to crop yield^44^. Li et al.^45^ showed that slow nitrogen release fertilizer could increase nitrogen uptake in the middle and late growth stages of corn.

When the plant is exposed to salt stress, nitrogen absorption decreases. The reduction of nitrogen absorption in saline conditions is due to the reduction of plant root permeability, reduction of soil microbial activity and reduction of nitrate absorption^46^. Oyinlola et al.^47^ investigated the growth of tomato plants in different soil textures (clay, loam, and sand) and different concentrations of nitrogen. The shoot total nitrogen in plants grown in the clay loam soil was more than the sandy loam soil.

### The effect of application of treatments on the absorbable potassium of aerial organs

**Figure 5:**
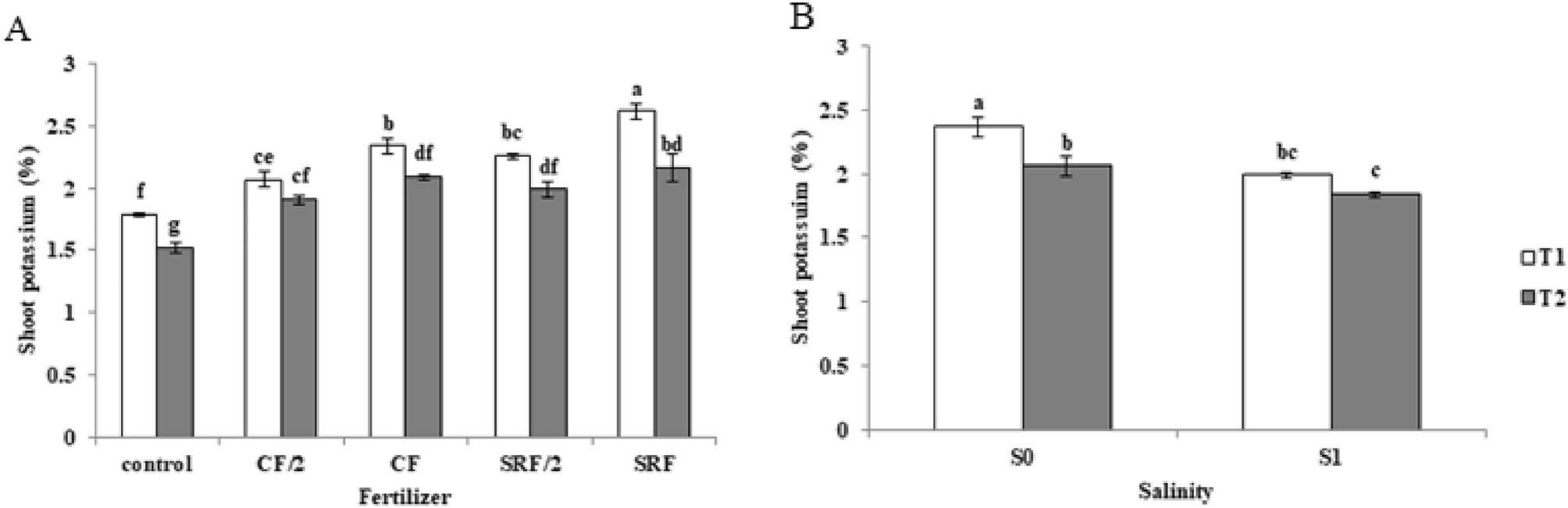
The effect of treatments on shoot potassium (**A**) the effect of soil salinity level and fertilizer treatments on the absorbable potassium of aerial organs, and (**B**) Tthe effect of soil salinity level and soil type on the absorbable potassium of aerial organs. Commercial fertilizer: (CF), half the amount of Commercial fertilizer used: (CF/2), slow -release fertilizer: (SRF), half the amount of slow -release fertilizer used: (SRF/2), non-saline soil: (S0), saline soil: (S1), Clay loam: (T1), Sandy loam: (T2).

The highest concentration of potassium that can be absorbed by aerial parts in the absence of salinity stress was recorded in the slow release fertilizer treatment, which is 2.79%, which increased by 46% compared to the control (Fig. 5).

Using potassium fertilizer increases the potassium solution in the soil and increases the concentration gradient, and diffusion of potassium to the root surface. As a result, more potassium is absorbed by the plant and its concentration increases in the plant tissue. Using of potassium can improve the stability of enzymes and proteins which subsequently lead to plant growth. Tian et al.^42^ investigated the effect of the slow-release potassium fertilizer on cotton plants, and their results showed that the absorbable soil potassium, chlorophyll index, and net photosynthesis rate (Pn), maximum photochemical efficiency (Fv/Fm) and effective quantum yield Photosystem (II) (PSII) increased. potassium addition during salt stress improved H + -ATPase activity in plasma-membranes, restored photosynthetic linear and cyclic electron flow, restored the activity of key Photosystem I (PSI) and PSII proteins, and increased dark-adapted PSII photochemical yield Fv/Fm, while reducing non-photochemical quenching. In this research, the concentration of potassium in the aerial parts decreased in saline soils. The increase of sodium in leaves under saline conditions and its toxic effects can be the direct effects of sodium toxicity due to the reduction of potassium and calcium nutrients^48,49^. A high concentration of sodium prevents the absorption of nitrogen and potassium nutrients^50^. Bazargan et al.^51^ investigated potassium in three types of soil texture. They stated that potassium in aerial parts was higher in soil with clay loam texture versus sandy soil. As the cation exchange capacity decreased (the soil texture became lighter), the concentration of potassium in the aerial parts of the plant decreased.

### The effect of the application of treatments on the phosphorus of aerial organs

**Figure 6:**
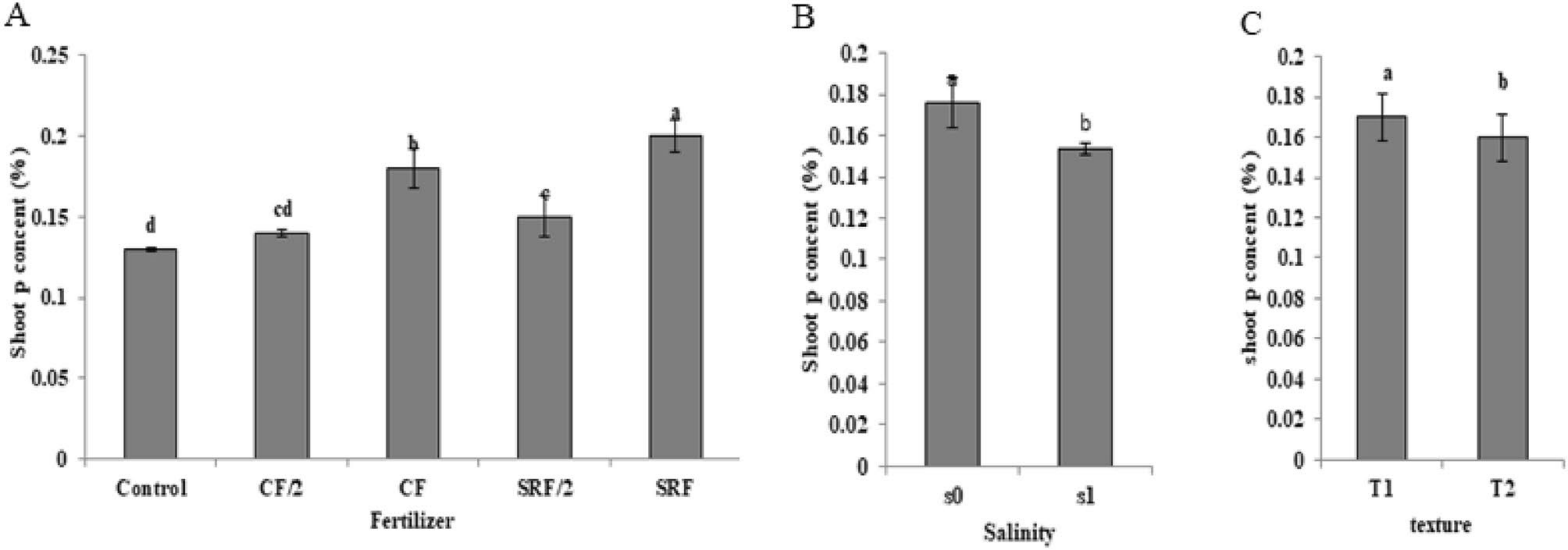
The effect of treatments on shoot phosphorus content (**A**) The effects of fertilizer on plant phosphorus, (**B**) The effects of salinity on plant potassium and (**C**) The effects of soil texture on plant phosphorus.

The maximum phosphorus content was observed in the SRF treatments. The highest phosphorus concentration in the aerial organs in SRF treatment was 0.2%, which was 66% more than the control (Fig. 6).

Commercial fertilizer: (CF), half the amount of commercial fertilizer used: (CF/2), slow- release fertilizer: (SRF), half the amount of slow -release fertilizer used: (SRF/2), non-saline soil: (S0), saline soil: (S1), Clay loam: (T1), Sandy loam: (T2).

The availability of phosphorus for absorption by the plant in the early stages of growth is critical to reach optimal yield^52^. A slow-release NPK fertilizer was prepared using potassium dihydrogen phosphate and urea fertilizers and evaluated in the soil^53^. The results showed 91.8% of soil phosphorus was released in the soil for 30 days. Salinity decreased the concentration of phosphorus in the plant. The maximum concentration of phosphorus was reported in non-saline soils and the minimum was in saline soils. Salinity reduces the absorption of phosphorus by the roots and its transfer from the roots to the shoots and the redistribution of phosphorus from old leaves to young leaves, which is due to the decrease in the mobility of phosphorus stored in vacuoles^54^. In the clay loam soils, the shoot phosphorus content was higher than the sandy loam. Zheng et al.^55^ investigated the effect of soil texture on fertilizer and soil phosphorus. They stated that in clay soils the plant phosphorus uptake at 80 mg P kg^–1^ of soil was higher than the coarser-textured soils (sandy loam, loam, and clay loam).

### Effect of the application of treatments on the iron of aerial organs

**Figure 7:**
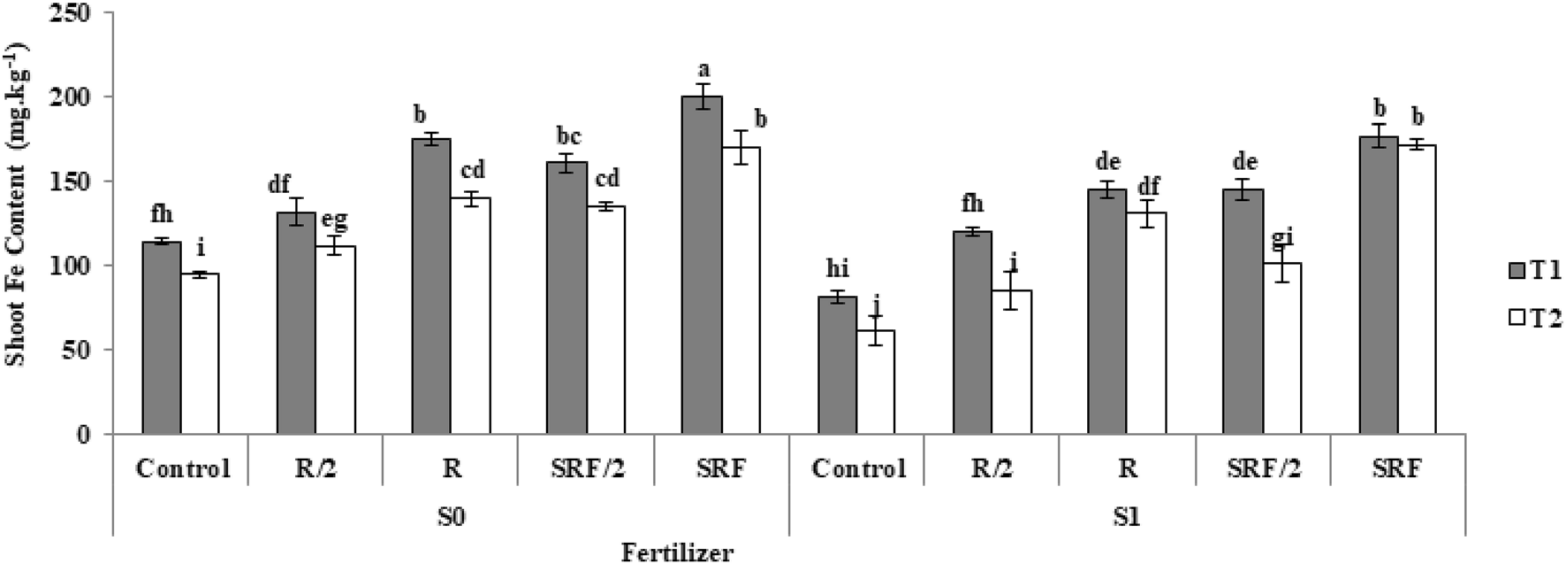
The effect of the application of treatments on shoot iron content commercial fertilizer: (CF), half the amount of commercial fertilizer used: (CF/2), slow-release fertilizer: (SRF), half the amount of slow- release fertilizer used: (SRF/2), non-saline soil: (S0), saline soil: (S1), Clay loam: (T1), Sandy loam: (T2).

Results of analysis variance (Table 2) showed that the interaction effects were significant at 1% (*p* ≤ 0.05). The highest amount of shoot Fe content was observed in the slow -release fertilizer treatment. The highest shoot Fe content in the aerial organs was 200 mg kg^−1^, which was 75% higher than control (Fig. 7).

Magnetite nanoparticles have a remarkable ability to extract total iron from the soil and transfer iron particles to the roots, stems, and leaves of the plant, which increases the movement of iron in the rhizosphere and increases the rate of iron absorption. Iron acts as an activator for biochemical processes such as respiration, photosynthesis and symbiotic nitrogen fixation, and protein metabolism involved in plant growth^56^.

Yan et al.^57^ investigated the effect of iron nanoparticles on the corn plant. The amount of iron in the plant tissue increased by 209% and 271% respectively in the presence of 50 and 500 mg kg^−1^ Fe_3_O_4_ NPs compared to the control. These results can be attributed to the function of the iron in cellular metabolism and to the role of enzymes in photosynthesis^58^. In this research, under salt treatment, a significant decrease in the content of iron in the aerial parts was observed. Anjum et al.^59^ reported that with an increase in salinity (80 mM), the concentration of iron, zinc, and copper elements in citrus leaves and roots decreases. The reason for the reduction of low-use elements in saline conditions can be the absorption of more elements such as sodium, magnesium, and calcium^60^.

### Effect of the application of treatments on the zinc of aerial organs

**Figure 8:**
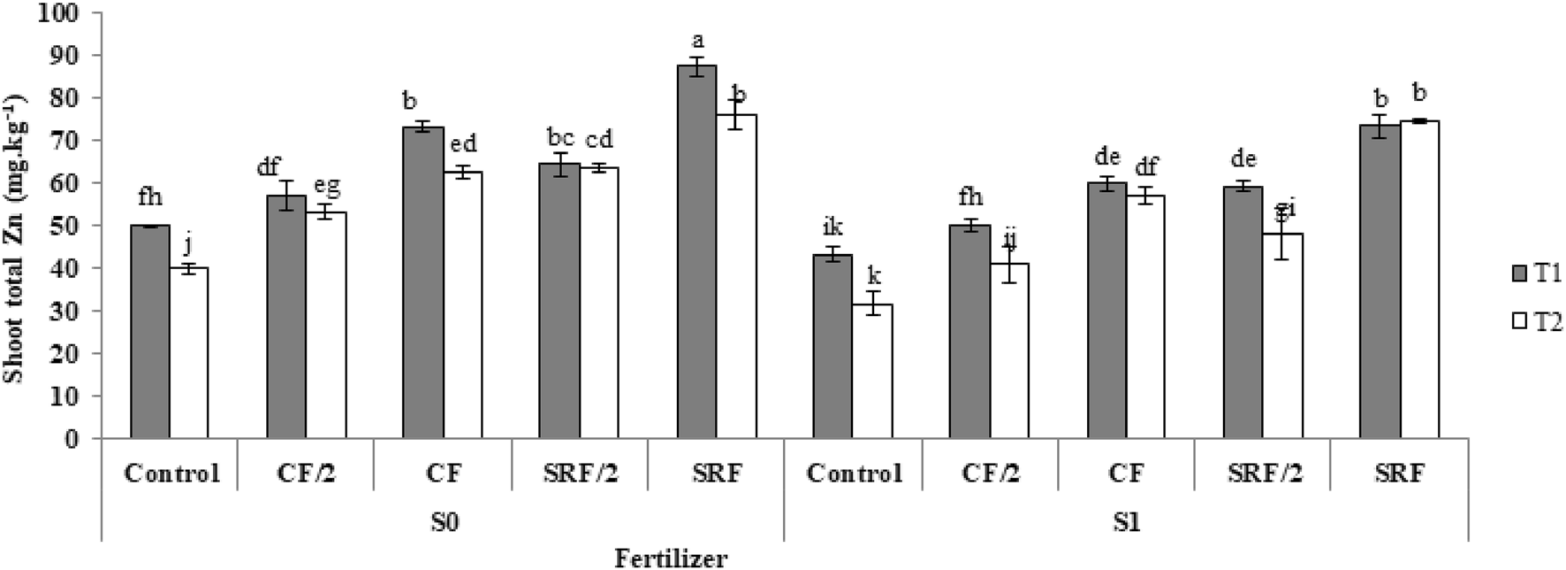
The effect of application of treatments on shoot zinc content commercial fertilizer: (CF), half the amount of Commercial fertilizer used: (CF/2), slow -release fertilizer: (SRF), half the amount of slow- release fertilizer used: (SRF/2), non-saline soil: (S0), saline soil: (S1), Clay loam: (T1), Sandy loam: (T2).

The highest amount of shoot total Zn was observed in the slow-release fertilizer treatment. The highest shoot total Zn concentration in the aerial organs was 87.41 mg kg^−1^, which was 74% higher than control (Fig. 8).

Plants need low-consumption elements for proper growth and normal metabolism. Many elements are also involved in the catalytic oxidation and reduction reactions of plants. A deficiency of low-use elements can lead to arrested growth and leaf necrosis due to elemental disturbances in various processes, such as electron transfer^34^. Many studies have been conducted that have stated that nanofertilizers increase the number of plant nutrients^61^. Deshpande et al.^62^ stated that zinc complex chitosan nanoparticles (Zn-CNP) act as a suitable nanocarrier for micronutrients for foliar application in wheat. Zn-CNP released the micronutrients slowly. The concentration of Zn in wheat germs increased by 27% in the variety (MACS 3125) and 42% in the variety (UC1114), indicating the appropriate use of chitosan-based nanoparticles in agricultural fertilizers. The increase in salinity caused a decrease in zinc in the aerial parts of the plant. At high salinity, bicarbonate, chlorine, and sulfate anions Zn mobility and reduce Zn availability for plants^63^. It has been previously reported that salinity decreased the zinc concentration in mango stem^64^ and jujube leaves and stems^65^.

## Conclusion

In this study, we have demonstrated the use of chitosan – nanographene oxide as a nutrient carrier, resulting in a slow -release and sustained delivery of Fe and Zn. The chitosan nanographene oxide GO-CS coating creates a potent complex with the fertilizer and leads to a slower-release of iron and zinc. The use of graphene oxide in nanocomposites may block some of the interconnected pores of chitosan (CS) and delay the release of zinc and iron.

In addition to having good controlled release properties, these coatings have various environmental and economic advantages such as renewability and biodegradability. In this study, polymer latex containing starch-g-poly and nano biochar (NCNP) was used to prepare NPK slow-release fertilizer which utilization of starch as a natural and environment friendly biopolymer and nano biochar in the synthesis of slow release fertilizer increased the efficiency of fertilizer. One of the advantages of this slow release fertilizer is using water instead of organic solvent. The use of slow-release fertilizers improved the plant growth parameters including the plant height and the plant fresh and dry weight, as well as the plant chlorophyll content. In general, the use of slow-release fertilizers reduces the amount of fertilizer consumption, prevents wastage of fertilizers and reduces environmental pollution.

The use of slow-release fertilizers increased total shoot nitrogen, shoot phosphorus, shoot potassium, shoot iron, and shoot zinc content by 44, 66, 46, 75, and 74% compared to control. The results also showed the use of synthetic slow-release fertilizers increases the absorption of nutrients by the plant, and improve plant growth. Also, synthetic fertilizers are low in price, good biodegradability. Nano-fertilizer can be introduced as a cheap, effective, and biocompatible option to solve iron and zinc deficiency in the soil. More complete research on these synthesized fertilizers is underway and will be tested in more extensive comparisons.

## Data Availability

The datasets used and/or analysed during the current study available from the corresponding author on reasonable request.
